# Facial Feminization Surgery: Anatomical Differences, Preoperative Planning, Techniques, and Ethical Considerations

**DOI:** 10.3390/medicina59122070

**Published:** 2023-11-24

**Authors:** Sarah L. Barnett, Joshua Choe, Christopher Aiello, James P. Bradley

**Affiliations:** 1 Perelman School of Medicine, University of Pennsylvania, Philadelphia, PA 19104, USA; sarahbarnett1999@gmail.com; 2 Northwell Health Division of Plastic Surgery, Zucker School of Medicine at Hofstra/Northwell, Lake Success, NY 11042, USA; joshua.choe00@gmail.com (J.C.); caiello@northwell.edu (C.A.)

**Keywords:** facial feminization surgery, virtual surgical planning, artificial intelligence, gender dysphoria

## Abstract

Facial Feminization Surgery (FFS) is a transformative surgical approach aimed at aligning the facial features of transgender women with their gender identity. Through a systematic analysis, this paper explores the clinical differences between male and female facial skeletons along with the craniofacial techniques employed in FFS for each region. The preoperative planning stage is highlighted, emphasizing the importance of virtual planning and AI morphing as valuable tools to be used to achieve surgical precision. Consideration is given to special circumstances, such as procedure sequencing for older patients and silicone removal. Clinical outcomes, through patient-reported outcome measures and AI-based gender-typing assessments, showcase the efficacy of FFS in achieving proper gender recognition and alleviating gender dysphoria. This comprehensive review not only offers valuable insights into the current state of knowledge regarding FFS but also emphasizes the potential of artificial intelligence in outcome evaluation and surgical planning to further advance patient care and satisfaction with FFS.

## 1. Introduction

Gender dysphoria is a condition in which transgender individuals experience a misalignment between their gender identity and their biological primary and secondary sexual characteristics [1]. The prevalence of gender dysphoria is estimated to be approximately one in 30,000 male-assigned births and one in 100,000 female-assigned births, as reported by the World Professional Association for Transgender Health [2]. Surgical intervention for gender dysphoria traditionally focused on genital reconstruction; that is, until 1983, when Dr. Douglas Ousterhout pioneered Facial Feminization Surgery (FFS) as a groundbreaking approach to address the facial discrepancies between male and female genders [3]. Because gender identity is deeply intertwined with facial appearance, FFS plays a crucial role in aligning a person’s external features with their internal identity.

Dr. Ousterhout, drawing upon extensive expertise in craniomaxillofacial, reconstructive, plastic, and aesthetic surgeries, aimed to explore the clinical differences between male and female facial skeletons through objective physical anthropological measurements [3]. As facial aesthetic parameters continually change, FFS techniques have evolved with the times, incorporating advancements in aesthetics, technology, and patient input [4].

Beyond its cosmetic implications, FFS represents a transformative procedure that alters the fundamental structure of the face, replicating the characteristic features associated with femininity. It targets both the bony framework and soft tissue elements, while hormone therapy primarily affects the latter while leaving the former relatively unchanged. By modifying the facial supportive skeleton, FFS aims to accentuate feminine facial traits and reduce the psychological burden of incongruence. This has been shown to lessen gender dysphoria, contributing to improved mental health and overall well-being for transgender women [5].

**Figure 1:**
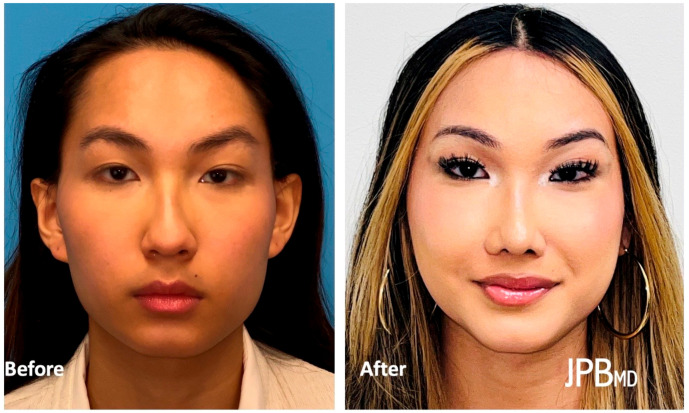
Before (**left**) and after (**right**) Facial Feminization Surgery after undergoing frontal sinus setback, supraorbital contouring, hairline lowering, brow lift, rhinoplasty, mandibular angle reduction, genioplasty, malar fat grafting, submental fat excision, and tracheal shave.

FFS typically encompasses a comprehensive set of procedures targeting the most sexually dimorphic facial regions. These include the forehead, orbits, nose, chin, jaw, and neck, which exhibit distinct differences between male and female individuals [6]. Male faces tend to feature square and angular contours, prominent jawlines, and chins, while female faces exhibit softer, more oval-shaped characteristics with delicate chins [7]. By employing a combination of surgical techniques such as frontal sinus setback, supraorbital contouring, hairline lowering, brow lift, rhinoplasty, mandibular angle reduction, genioplasty, malar fat grafting, fat removal, upper lip lift, lip augmentation, and tracheal shave, FFS aims to achieve a more feminine appearance [8] (Figure 1).

In this review paper, we will explore the different facial regions that are commonly addressed in FFS procedures and delve into the preoperative planning, perioperative care, clinical outcomes, and future directions of this transformative surgical approach. By synthesizing the findings from relevant studies, we aim to provide a comprehensive overview of the current state of knowledge in the field of Facial Feminization Surgery.

## 2. Key Anatomical Differences between Male and Female Facial Regions and Surgical Modification

### 2.1. Forehead/Brow

The forehead and brow region exhibit significant differences between males and females in both skeletal and soft tissue features. Males tend to have a longer forehead with a heavier and horizontally positioned brow on the superior orbital rim [9]. In contrast, females have a shorter forehead, an arched and rising brow, and an “upside-down U shape” hairline. Hairline lowering and brow lift procedures address these soft tissue differences [7]. Surgical techniques such as burring, frontal sinus setback, and supraorbital contouring are used to modify the skeletal forehead/brow, depending on the anatomical severity [10].

### 2.2. Jawline

The jawline is primarily determined by the mandible and chin. Male mandibles have a more prominent flare, a thicker external oblique ridge, and larger condyles [9,11]. Based on the degree of lower face width, various techniques such as serial Botox injections, masseter resection, mandibular angle resection, inferior border resection, mandibular shaving, and counterclockwise rotational double jaw surgery can be employed to create a more feminine jawline. Chin feminization involves chin width reduction through burring or osseous genioplasty narrowing, along with vertical shortening and advancement if necessary [11,12,13].

### 2.3. Neck

The neck often exhibits masculine features such as the presence of the “Adam’s apple” due to the acute angle of the superior thyroid cartilage in males [8]. Tracheal shave, or chondrolaryngoplasty, is commonly performed to feminize the neck by reducing the prominence of the thyroid cartilage [14]. Submental fat excision and platysmaplasty tightening can also be performed to further enhance the desired neck silhouette. These three procedures can be performed in a single-stage reconstruction, through the same incision made discreetly in the submental region [10].

### 2.4. Nose

Male and female noses differ in terms of bony and cartilaginous structure. Male nasal bones are larger with a higher aperture, resulting in an acute glabellar angle and a more prominent dorsal hump [12,14]. In contrast, the female nasal dorsum is concave, and the nasal tip is upturned, contributing to a more obtuse nasolabial angle [11,15,16,17]. Differences in nasal size and shape can be addressed through rhinoplasty and frontal bossing techniques, taking into account ethnic variations and modification of the nasofrontal junction.

### 2.5. Lips

Subtle differences exist between male and female lips, including the visibility of the dry vermillion, the shape of the cupid’s bow, and the distance from the nasal base to the vermillion border. Male lips are typically thinner, with less maxillary incisor and gingival display [8]. Lip feminization techniques involve upper lip lifts to expose more of the incisor teeth at repose as well as lip augmentation using fillers, fat, tendon, or fascia to achieve a fuller appearance.

### 2.6. Cheeks

The malar soft tissues and zygomatic bones contribute to differences in male and female cheeks. Males have flatter zygomas with less projection, resulting in less triangulation of the face [11]. The triangulation is dependent on points between the cheek and chin, where the chin is the apex of the triangle, and the lateral points of the cheeks form the base of the triangle [8]. Female cheeks are higher, more anteriorly positioned, and may exhibit hollowness inferiorly [7]. Augmentation of the malar region can be achieved through structured fat grafting or implants, while buccal fat removal can create lower cheek hollowing. Zygomatic arch repositioning has been shown to be beneficial to reduce face width in some Asian patients [10].

## 3. Preoperative Planning

### 3.1. Virtual Planning

**Figure 2:**
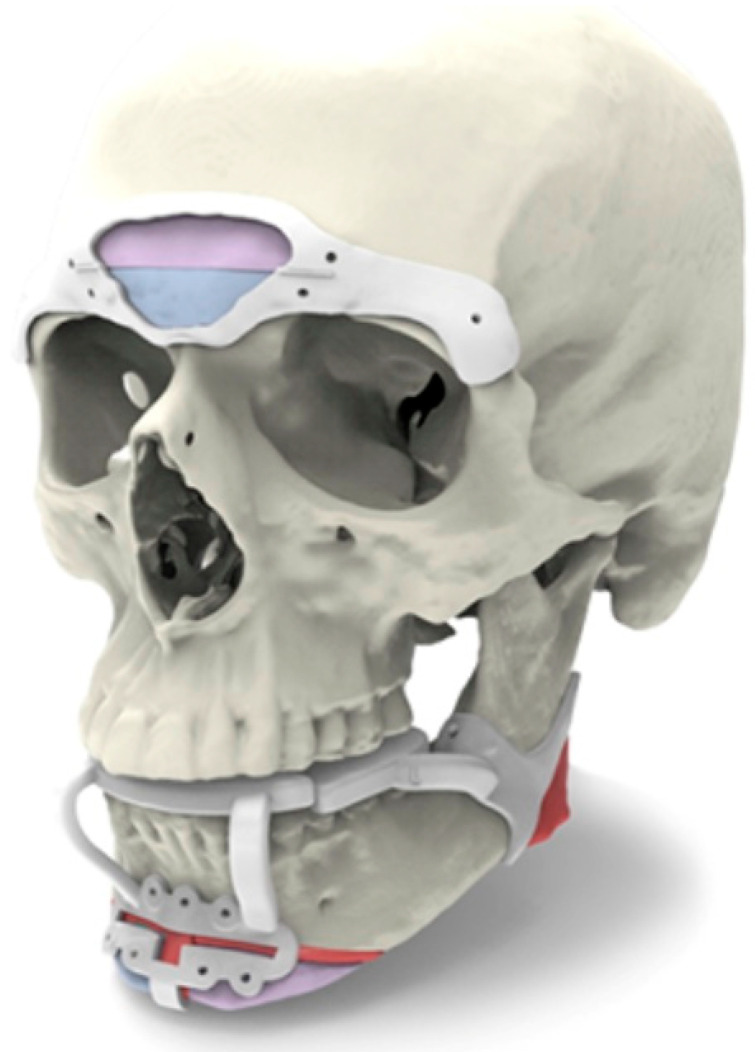
Virtual Surgical Planning for the brow, jawline, and chin regions. A frontal brow cutting guide is used for osteotomy of the anterior frontal sinus. A lateral brow cutting guide is used for osteotomy of the lateral supraorbital bar. Cutting guides may also be used for reduction, advancement, and narrowing of the chin along with mandibular angle resection.

Preoperative virtual planning has emerged as an essential tool in FFS (Figure 2). The utilization of cutting guides and advanced imaging techniques allows surgeons to precisely visualize the desired facial changes and plan the surgical procedures accordingly. Computer-aided design (CAD) and computer-aided manufacturing (CAM) have demonstrated benefits in orthognathic surgery and oncologic jaw reconstruction. Similarly, these technologies have proven helpful in virtual surgical planning for FFS [18,19]. Computed tomographic (CT) scans provide detailed three-dimensional anatomical information for morphologic typing and the creation of cutting guides and custom plates, optimizing accuracy, safety, efficiency, and predictability. Preoperative virtual planning is commonly utilized for anterior frontal sinus wall setback, lateral supraorbital recontouring, osseous genioplasty, and mandibular angle reduction [7,20].

Although the traditional approach without virtual planning remains valid, the use of cutting guides and custom plates can reduce operative time and increase precision. Studies have shown decreased operative time and improved control with the use of cutting guides for anterior frontal sinus wall setback [7]. Virtual planning also minimizes the risk of errors and complications, such as over-resection or nerve injury [21]. At the time of virtual planning, it is important to consider the differences in facial proportions that exist between males and females [22]. Additionally, virtual planning allows patients to actively participate in the preoperative process, although surgeons must ensure realistic expectations for optimal patient satisfaction.

### 3.2. Artificial Intelligence Preoperative Morphing

**Figure 3:**
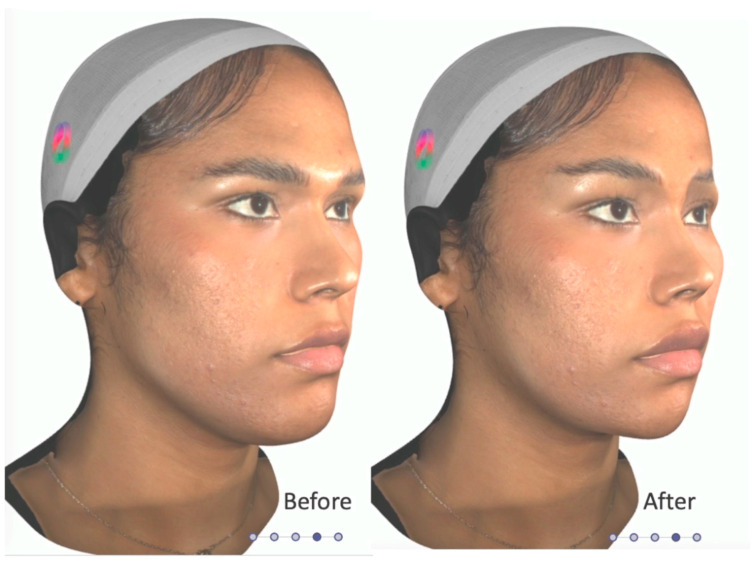
Artificial Intelligence-based 3D morphing software (version 2.0.0) used to make real-time adjustments for each facial region involved in FFS. Oblique view before and after morphing of the brow, nose, jawline, and lip regions.

Aligning patient expectations with realistic surgical outcomes is a mainstay in plastic surgery. Throughout history, surgeons have worked towards this goal with the use of simplistic illustrations that attempt to display the expected surgical outcome. Artificial intelligence (AI) has the potential to revolutionize the process of morphing patient images. Many companies have developed handheld camera systems that possess advanced technical capabilities with straightforward user interfaces [23]. This technology can be applied to the preoperative FFS visit to generate three-dimensional (3D) morphs of the patient’s face. These morphs can then be adjusted in real-time to show patients the potential outcomes of FFS operations on their own faces. The software company DeepSurface AI (version 2.0.0) has developed AI specifically for preoperative morphing for FFS patients. This software can be used to grossly augment each facial region involved in FFS by dragging an icon across the feminine–masculine spectrum. More fine-tuned adjustments can also be performed (for example, an isolated reduction of the dorsal bump while leaving the rest of the nose untouched) [24] (Figure 3).

AI morphing before FFS provides benefits to both patients and providers. When patients can visualize potential surgical changes on their own faces, instead of in abstract terms, they can more effectively communicate their goals and preferences. This precision provides the surgeon an opportunity to align patient goals with what can realistically be accomplished in their surgery. Studies have shown that patients have a positive perception and satisfaction with photographic techniques used to predict the outcome of aesthetic procedures [25]. 3D morphs have been shown to generate models that can be measured within clinical limits. AI predictions of breast reconstruction procedures matched closely with postoperative outcomes [26,27]. AI morphing has not yet been studied in the context of FFS. While preliminary results seem promising, there remains uncertainty about the use of this software for FFS. One potential risk is that the technology may be counterproductive to managing patient expectations. For example, if patients expect exactly what they are shown in preoperative morphs but inevitable surgical variability alters outcomes away from the model. To this point, some studies indicate that measurements taken from morphs can occasionally be outside of clinical limits [28]. In this way, AI morphing has the potential to decrease patient satisfaction. One breast surgeon found a nonsignificant difference when comparing satisfaction scores between patients who used 3D imaging to predict outcomes preoperatively versus those who did not [29]. Cautious use and more research are indicated to better inform the use of AI technology in the setting of FFS.

## 4. General Craniofacial Techniques by Region

### 4.1. Brow

**Figure 4:**
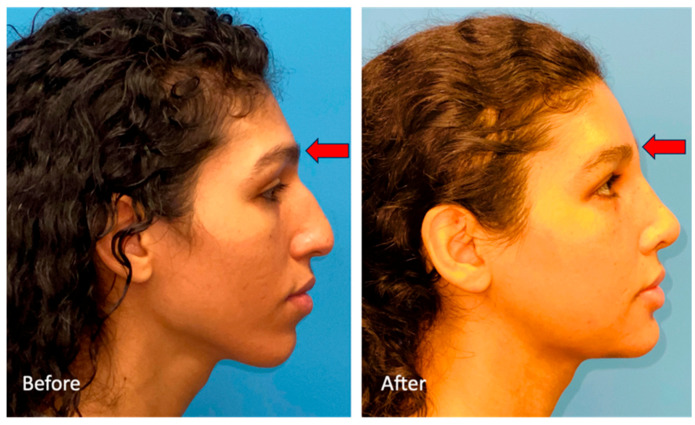
Lateral view of a patient with a type 3 frontal central brow before (**left**) and after (**right**) FFS. Before FFS, this patient displayed bossing and large projection of the anterior table. The change in profile appearance is especially notable when looking at the nasofrontal junction (red arrow). This patient also underwent mandibular angle reduction and osseous genioplasty using custom cutting guides.

The modification process for the forehead and brow regions begins with a zig-zag coronal incision, supplemented by an anterior pretrichial incision if hairline lowering is needed. To protect the frontal branch of the facial nerve, a dissection of the deep temporal fascial plane is conducted laterally, in tandem with a central subgaleal and then subperiosteal dissection [30]. For frontal sinus setback, a cutting guide—developed from detailed virtual planning—outlines the anterior wall border and septum(s). Following osteotomy using a Midas Rex B1 drill, the anterior wall is reshaped and secured with a resorbable plate, modifying the overall forehead contour (Figure 4). Closure is meticulously performed in layers over a drain to ensure optimal healing and aesthetic outcomes.

### 4.2. Jawline

Feminization of the lower face commonly involves the reduction, narrowing, and advancement of the chin, accomplished through a carefully placed transverse incision below the mucogingival junction [31]. Genioplasty cutting guides and custom plates are utilized to ensure precise reshaping of the chin. Similarly, mandibular angle reduction is performed through a lateral gingivobuccal sulcus incision. 3D models can be used for verification of the size of the removed bone when applicable [10]. This approach allows for precise osteotomy, contouring, and ultimately, an aesthetically pleasing outcome. Attention to detail and inferior alveolar nerve localization using preoperative CT scans are crucial during this procedure to ensure proper healing and to minimize potential postoperative complications.

### 4.3. Neck

Feminization of the neck includes laryngotracheal reduction performed through a transverse incision at the midpoint between the submental crease and the cervicomental angle [8]. Submental and subplatysmal fat may be excised as needed during dissection. A V-shaped segment of the thyroid cartilage is removed. A rim of cartilage superior to the level of the vocal cords is preserved for stabilization [17,32]. Finally, platysma muscle unification and removal of excess skin are performed to maximize neck tightening.

### 4.4. Nose

**Figure 5:**
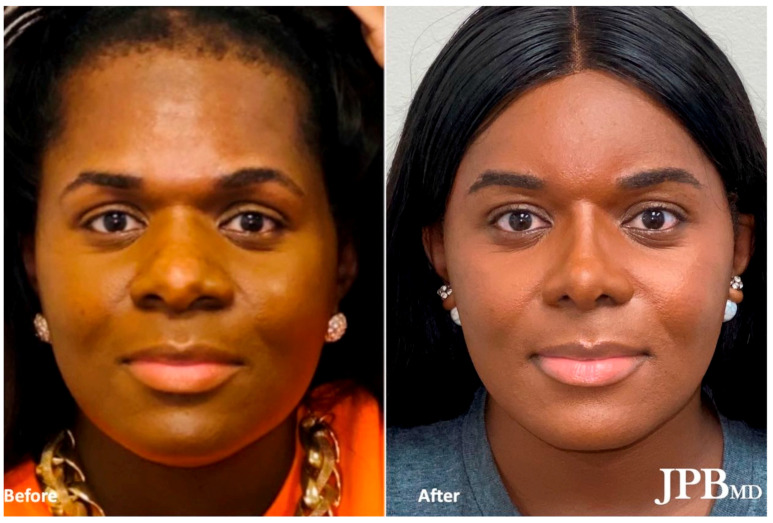
Frontal view of a patient who underwent feminization rhinoplasty including alar base reduction to decrease alar base width and nostril size. This patient also shows a significant narrowing of both the dorsal bridge and tip width.

The feminization rhinoplasty approach encapsulates a variety of techniques such as dorsal hump reduction, caudal septal trim for upward tip rotation, and alar base reduction [33]. Using an open approach to provide optimal access and visibility, the procedural sequence includes mid-columellar stair-step and marginal incisions, cephalic scroll resection, creation of bilateral spreader graft pockets, and septal resection. The removed cartilage is repurposed into grafts that are securely placed for improved aesthetics, but allograft cadaveric cartilage may also be used. Further, alar base resection is selectively employed to reduce nostril size and correct asymmetries, followed by lateral nasal bone infracturing to narrow the middle vault [34,35] (Figure 5).

### 4.5. Lips

Lip augmentation can be achieved in the office using hyaluronic acid dermal filler injections, or more permanently, in the operating room using various autologous materials such as rolled-up temporal fascia or palmaris longus tendon [36]. In cases where an upper lip lift is desired, “bullhorn” or “gull-wing” subnasal excision techniques can be used to enhance dental show [37]. Lip lifts are often staged separately from open rhinoplasty procedures.

### 4.6. Cheeks

Cheek feminization may involve fat grafting, often harvested from the abdomen, to create a fuller and rounder appearance [38]. Implants may also be used for cheek augmentation. Concurrently, intraoral buccal fat excision is performed to reduce lateral fullness. In cases where facial width reduction is desired, zygomatic arch infracturing is executed.

## 5. Special Circumstances

### 5.1. Procedure Sequence and Timing

A well-organized sequence of FFS procedures ensures patient safety and optimal surgical outcomes. FFS can be approached using multiple methods, either as a comprehensive single-stage procedure or through sequential stages [35]. Completing multiple facial feminization procedures in a single operation offers convenience to patients while minimizing overall recovery time [39]. However, patient safety must be prioritized by considering any co-existing medical conditions as well as the time required to complete the procedures. Notably, a study by Chaya et al. found that an increased number of procedures performed per anesthetic event in FFS patients did not predict higher complication rates [40]. Hence, patients can safely undergo multiple facial feminization procedures simultaneously under appropriate medical judgment. The “all-in-one” approach is often favored due to demonstrated increases in patient satisfaction rates and cost savings [41,42].

### 5.2. The Older Patient

An alternative staging strategy may be preferred in aging patients. This involves initially focusing on structural hard tissue procedures such as frontal sinus setback, supraorbital contouring, jawline tapering, and rhinoplasty, followed by subsequent soft tissue procedures such as facelift, neck lift, and blepharoplasty. This approach is analogous to restructuring the foundation (hard tissue) before the house (soft tissue). Older patients may require additional cosmetic procedures after bone modification due to factors such as facial sagging and skin laxity. For example, because all suprahyoid muscles attach at the lower mandible border, shaving the jaw will result in laxity of the muscles and skin, more significantly in older patients with preexisting laxity [16]. This can only be corrected through a neck lift. Comprehensive assessments and appropriate surgical interventions are essential for achieving satisfactory outcomes in this patient population.

### 5.3. Silicone Removal

Silicone is often used in facial feminization to augment areas such as the cheeks, chin, and jawline, and to provide patients with a more feminine facial contour. Over time, some patients may decide to have these implants removed due to various reasons, including changes in aesthetic preferences or concerns regarding long-term implant safety and complications. The use of silicone, whether implants or injectable, for soft tissue augmentation is controversial. Injectable silicone has historically been illegally obtained and used incorrectly by nonmedical practitioners, causing significant harm to patients. Even when used correctly, serious adverse events have been reported, including granulomatous nodules, ulceration, and cellulitis [43]. When removal is indicated for any reason, surgical excision may be employed. However, this is complicated by the permanence of the silicone filler and its tendency to migrate to other parts of the body [44]. The decision to remove silicone implants can significantly impact subsequent FFS procedures, as it alters the soft tissue appearance and malleability, and necessitates consideration of alternative techniques such as fat grafting to achieve the desired feminine facial appearance.

## 6. Perioperative Care

During the initial consultation, pertinent information regarding the patient’s transition timeline and duration of hormone replacement therapy (HRT) is collected. It is important to note that the feminization of facial features, including soft tissue, skin, fat, and hair, can be observed with HRT. Consequently, it is recommended that patients allow a minimum of 12–18 months of HRT prior to undergoing FFS. Following a comprehensive facial examination and discussion of the patient’s concerns, potential FFS procedures for modifying facial features are explored. To facilitate insurance submission, a letter of support from the patient’s hormone provider and mental health therapist is required. Preoperative virtual planning involves obtaining a maxillofacial CT scan with 3D reconstruction [10].

During the subsequent preoperative appointment, the specific FFS procedures and associated risks are thoroughly discussed. Within 30 days prior to surgery, patients are required to consult their primary care provider for necessary pre-surgical testing and clearance. Pain management strategies are carefully addressed to minimize opioid use, which may involve medications such as Acetaminophen, Gabapentin, and NSAIDs. It is strongly advised that patients abstain from smoking marijuana, cigarettes, or vaporizers for at least 3 weeks before and 4 weeks following surgery to promote optimal healing [10].

Depending on the number of procedures indicated, FFS typically takes 6–8 h, with a standard two-night hospital stay. At the time of discharge, the surgical dressing and drain are replaced with a jaw bra, which is to be worn continuously for 2 weeks and then during the night for the subsequent 6 weeks. Patients are advised to adhere to a soft diet for 2 weeks and to utilize Peridex mouthwash after meals. To minimize swelling, patients are instructed to keep their heads elevated at a 30-degree angle. Postoperative follow-up appointments are scheduled weekly for the first 3 weeks and then every 3 months during the initial year, as final results may take several months to manifest [10].

## 7. Clinical Outcomes

The primary objectives of facial feminization surgery are twofold: firstly, to ensure transwomen are recognized as female in social settings, and secondly, to alleviate gender dysphoria and enhance patient satisfaction. Various methods have been employed to evaluate the success of FFS in accomplishing these goals, including artificial intelligence, crowdsourcing, and patient-reported outcome measures.

In a study by Raffaini et al., patient satisfaction was evaluated using a questionnaire, revealing notable improvements in the physical, mental, and social aspects of life following FFS [42]. At the societal level, a public-opinion crowdsourcing study highlighted that transgender women utilizing hormonal therapy, make-up, and hairstyles for feminization were still misgendered nearly half of the time. However, after undergoing FFS, patients were substantially more likely to be confidently identified as female by the public [45]. More objective outcome data was produced from neural networks trained to identify gender based on facial features. Preoperative images were misclassified by neural networks approximately half of the time, yet postoperative images were correctly identified as female at rates similar to cis-females (97%). This indicated a significant 45 percent improvement in gender-typing accuracy compared to preoperative images [46]. Together, the outcome measures employed in these studies demonstrate the success of FFS at the individual, societal, and clinical levels.

Similar methodologies should be employed to further refine surgical approaches. For example, gender-typing by neural networks and crowdsourcing could help elucidate which of the many FFS procedures produces the most significant effect on gender perception. The integration of artificial intelligence in assessing patient outcomes and predicting surgical results holds great potential for the future of FFS. AI algorithms can analyze large datasets to identify patterns, refine surgical techniques, and improve patient satisfaction. Further research and development in this area can revolutionize the field of FFS and enhance the overall patient experience.

## 8. Future Perspectives

As the interest and demand for FFS continues to rise, it is important to continue to perfect surgical techniques and perioperative care [47]. As aesthetic parameters and patient goals evolve with the times, it is important that surgeons continually adjust their techniques [4]. Similarly, as technology continues to evolve and the capabilities of AI programs broaden, these elements should be considered in the pre-surgical planning process. Surgeons should understand the evolving nature of FFS and be prepared for continuing improvements to this operation in the years to come.

## 9. Ethical Considerations

There are many legal and ethical considerations that should be taken into account in relation to FFS. These include but are not limited to societal constructs and gender, medical necessity, barriers to access, irreversibility, age of consent, femininity, and beauty. While FFS may be associated with legal or ethical controversy, research and physician experience recognizes FFS as medically necessary for individuals with gender dysphoria [48]. With access to FFS, individuals with gender dysphoria may show significant improvements in overall quality of life.

## 10. Conclusions

Facial Feminization Surgery is a transformative and highly individualized approach to aligning a person’s external appearance with their gender identity. This comprehensive review has highlighted the differences between male and female facial regions, preoperative planning techniques, craniofacial techniques employed in FFS, perioperative care, clinical outcomes, and future directions. The integration of preoperative advanced imaging with innovative surgical approaches and instrumentation has significantly improved outcomes and patient satisfaction. The continued advancements in FFS techniques, combined with the potential of AI morphing and analysis, hold promise for enhancing the well-being and quality of life of transgender individuals seeking facial feminization.

## Data Availability

No new data were created or analyzed in this study. Data sharing is not applicable to this article.
